# Exploring the therapeutic potential of phytochemicals apigenin and ellagic acid in managing polycystic ovarian syndrome and its comorbidities: a comprehensive review

**DOI:** 10.3389/fendo.2025.1633377

**Published:** 2025-11-05

**Authors:** Acharya Balkrishna, Maneesha Rana, Shalini Mishra, Ritik Agrawal, Satyendra Kumar Rajput, Muralikrishnan Dhanasekaran, Mamta Rana, Vedpriya Arya, Ramith Ramu, Ashutosh Upadhayay, Shalini Singh

**Affiliations:** ^1^ Patanjali Herbal Research Department, Patanjali Research Foundation, Haridwar, India; ^2^ Institute of Pharmaceutical Research, GLA University, Mathura, India; ^3^ Department of Pharmaceutical Sciences, Gurukul Kangri (Deemed to be University), Haridwar, India; ^4^ Department of Drug Discovery and Development, Harrison College of Pharmacy, Auburn University, Auburn, AL, United States; ^5^ Department of Yoga Science, University of Patanjali, Haridwar, India; ^6^ Department of Biotechnology and Bioinformatics, Academy of Higher Education and Research, Mysore, India; ^7^ School of Pharmaceutical Sciences, MVN University, Palwal, India

**Keywords:** PCOS, flavonoids, apigenin, ellagic acid, natural bio-actives, pharmacokinetics

## Abstract

Polycystic Ovarian Syndrome (PCOS) is a complex endocrine and metabolic disorder affecting women of reproductive age, characterized by hyperandrogenism, insulin resistance, chronic inflammation, and ovulatory dysfunction. Conventional therapies, such as oral contraceptives, insulin sensitizers, and anti-androgens, primarily offer symptomatic relief and are often associated with chronic adverse effects, underscoring the need for safer and more holistic alternatives. Naturally occurring bioactives have emerged as promising adjunct or alternative therapeutic agents in this context. This review critically examines the therapeutic potential of two phytochemicals or natural bioactives, apigenin and ellagic acid, in the integrative management of PCOS and its associated metabolic disturbances and comorbidities. Apigenin, a flavonoid abundantly present in parsley, chamomile, and citrus fruits, and ellagic acid, a polyphenol found in pomegranates and berries, both demonstrate significant anti-inflammatory, antioxidant, insulin-sensitizing, and anti-androgenic activities. Mechanistic studies reveal their ability to regulate ovarian steroidogenesis, suppress pro-inflammatory cytokines, improve insulin sensitivity via the PI3K/Akt signaling pathway, and reduce hyperandrogenism by inhibiting 5α-reductase. Preclinical and preliminary clinical studies support the efficacy of these treatments in restoring ovarian morphology, normalizing hormonal profiles, and ameliorating metabolic dysfunctions in PCOS models. Although limited by poor bioavailability, both compounds exhibit favorable safety and metabolic profiles, and emerging formulation approaches such as nano-delivery systems, phytosomes, and liposomes offer promising strategies to enhance their clinical applicability. This review advocates incorporating apigenin and ellagic acid into integrative PCOS treatment strategies. It highlights the need for well-designed clinical trials to validate efficacy, establish standardized dosing, and develop advanced delivery systems.

## Graphical Abstract

This graphical abstract represents an overview of PCOS pathology and the therapeutic potential of natural compounds apigenin and ellagic acid, highlighting their effects on androgen levels, insulin sensitivity, oxidative stress, and inflammation, with associated clinical and pre-clinical outcomes.

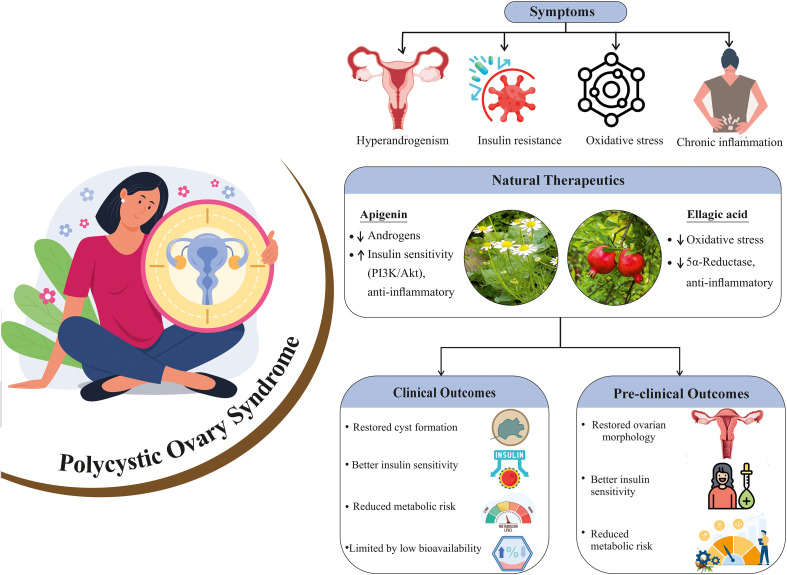

## 1 Background

Polycystic ovary syndrome (PCOS) is a prevalent endocrine and metabolic disorder affecting 4–20% of women of reproductive age worldwide, depending on diagnostic criteria (1). It is a complex condition marked by hormonal imbalance, insulin resistance, chronic low-grade inflammation, and reproductive dysfunction (2). These mechanisms underlie its clinical manifestations, which include menstrual irregularities, hyperandrogenism (hirsutism, acne, alopecia), anovulation, infertility, obesity, dyslipidemia, and type 2 diabetes (3). Due to its heterogeneous nature, PCOS presents diagnostic challenges and requires multidimensional management. Conventional treatments—oral contraceptives, metformin, and anti-androgens—remain first-line options but primarily manage symptoms without addressing root causes. Their use is further limited by adverse effects, hypersensitivity, and long-term dependency risks (4). For instance, spironolactone and metformin improve insulin resistance but cause side effects (5), while metformin may induce ovarian histological alterations (6). A meta-analysis also linked standard therapies to gastrointestinal intolerance, menstrual irregularities, and hepatotoxicity (7). These limitations have driven interest in safer, holistic alternatives, particularly natural bioactives with antioxidant, anti-inflammatory, and hormone-modulating properties (8). Evidence supports the efficacy of phytochemicals and medicinal plants (9), such as Mentha spicata, which improves hormonal balance and folliculogenesis (10). Among natural compounds, apigenin and ellagic acid have emerged as promising candidates (11). Apigenin, a flavonoid abundant in parsley, chamomile, celery, and citrus fruits, shows antioxidant, anti-inflammatory, anti-androgenic, and insulin-sensitizing properties (12). Preclinical studies report its ability to modulate ovarian steroidogenesis, enhance insulin sensitivity, and restore reproductive-metabolic balance (13). Similarly, ellagic acid, found in pomegranates, berries, nuts, and grapes, exerts insulin-sensitizing, anti-inflammatory, and anti-hyperandrogenic effects, while also improving ovarian function (14, 15). Both compounds reduce oxidative stress and key metabolic disturbances, potentially lowering long-term risks such as cardiovascular disease, hyperglycemia, and infertility. Beyond health implications, PCOS imposes a significant socioeconomic burden. Women often face reduced quality of life due to infertility, hirsutism, acne, obesity, and associated psychological distress, including anxiety and depression (16). These challenges increase healthcare utilization and treatment costs for metabolic comorbidities and infertility. In India, high prevalence is compounded by underdiagnosis, delayed care, and limited specialized infrastructure (17, 18). Globally, undiagnosed cases further magnify the burden (19). Given these concerns, exploring safe, effective, and targeted therapies is imperative. Natural bioactives such as apigenin and ellagic acid offer promising complementary or adjunct strategies to conventional treatments. This review evaluates their pharmacological properties, preclinical and clinical evidence, and integration potential into holistic treatment approaches, highlighting their role in improving reproductive, metabolic, and overall health outcomes in PCOS.

## 2 Methods

### 2.1 Search strategy and databases

A structured literature search was conducted across PubMed and Google Scholar to identify studies examining the therapeutic role of apigenin and ellagic acid in polycystic ovarian syndrome (PCOS). The search covered the period January 1999 to August 2025, corresponding to the timeframe of submission of this manuscript. The start year 1999 was selected as it marks the period following the widespread adoption of the Rotterdam and NIH criteria for PCOS diagnosis, which provided greater consistency in defining study populations. This timeframe also captures the rise of phytochemical and polyphenol research in endocrine and metabolic disorders, ensuring the inclusion of both foundational and contemporary evidence.

### 2.2 Search terms

We used controlled vocabulary and free-text terms in various combinations, including: “PCOS,” “polycystic ovary syndrome,” “apigenin,” “ellagic acid,” “flavonoids,” “polyphenols,” “natural bioactives,” “antioxidant,” “anti-inflammatory,” and “insulin sensitizer.” Boolean operators (AND/OR) were applied to refine the search.

### 2.3 Eligibility criteria

Inclusion: Preclinical (*in vitro* and *in vivo*) and clinical studies evaluating apigenin and/or ellagic acid in PCOS or related metabolic/endocrine dysfunctions; studies published in English; peer-reviewed full-text articles. Exclusion: Duplicates, conference abstracts without full data, editorials, commentaries, and studies not directly related to PCOS.

### 2.4 Study selection

The initial search (January 1999–August 2025) yielded 18,200 citations; after duplicate removal, 17,000 unique records remained. Titles and abstracts were screened independently by two reviewers, leading to 780 full-text articles retrieved for detailed evaluation. After applying eligibility criteria and excluding studies with methodological limitations or insufficient relevance, 107 articles were included in this review.

### 2.5 Quality appraisal

Although this is primarily a narrative review, the included studies were evaluated for methodological quality. Preclinical studies were assessed for reproducibility of models, dosing regimens, and outcome measures, while clinical studies were examined for study design (randomized, controlled, blinded), sample size, and risk of bias. The review was conducted under the PRISMA (Preferred Reporting Items for Systematic Reviews and Meta-Analyses) guidelines, where applicable, to ensure transparency and reproducibility. The search cutoff was August 2025, corresponding to the manuscript submission date, to ensure inclusion of the most up-to-date available evidence.

## 3 Global burdens of PCOS

The global prevalence of PCOS varies considerably by region, influenced by genetic predispositions, lifestyle and environmental factors, and the efficiency of healthcare delivery systems. A 2019 global survey highlighted marked geographic disparities, with the highest prevalence reported in countries such as Italy, Japan, and New Zealand, and the lowest in Bosnia, Albania, and North Macedonia (20). In South Asia, particularly in the Indian subcontinent, PCOS has emerged as a major public health challenge, with reported prevalence rates ranging from 9% to 22% among women of reproductive age (21). The burden is particularly pronounced in urban and semi-urban regions, where rapid transitions in lifestyle, characterized by poor dietary patterns, physical inactivity, obesity, and chronic psychosocial stress, are prevalent. Current estimates suggest that up to 20% of Indian women may be affected by PCOS, although underdiagnosis remains widespread due to low awareness, cultural stigma, and limited access to specialized care.

PCOS is among the most prevalent endocrine disorders in women, affecting approximately 8–13% of women of reproductive age worldwide (22). Alarmingly, up to 70% of cases remain undiagnosed, largely due to the lack of uniform diagnostic criteria, limited public awareness, and disparities in access to healthcare services (19). The choice of diagnostic criteria—NIH (1990), Rotterdam (2003), or AES (2006)—substantially influences reported prevalence. The NIH criteria are more restrictive, requiring both hyperandrogenism and oligo-anovulation, typically yielding lower prevalence rates (around 6–9%). The Rotterdam criteria, which require any two of three features (hyperandrogenism, oligo-anovulation, or polycystic ovarian morphology), generate higher prevalence estimates (up to 20%). The AES criteria, which prioritize hyperandrogenism, fall between these two approaches. This diagnostic variability complicates comparisons across populations and highlights the importance of standardized definitions for epidemiological surveillance. PCOS significantly impacts both reproductive and metabolic health across a broad age spectrum. Reproductive manifestations typically include infertility, menstrual irregularities, and hirsutism, while long-term complications often involve type 2 diabetes mellitus, cardiovascular disease, and an increased risk of endometrial cancer (23). In adolescents, early signs such as acne, irregular menstruation, and weight gain are frequently overlooked or misattributed, contributing to delays in diagnosis and intervention. The psychological burden of PCOS is also considerable. In sociocultural contexts such as India—where fertility and physical appearance are closely tied to identity—many women experience psychological distress, including anxiety, depression, low self-esteem, and body image dissatisfaction (16). Beyond health and psychological consequences, PCOS also imposes substantial economic costs. In the United States, the annual healthcare expenditure related to PCOS, including diagnosis, management of infertility, and treatment of comorbidities, has been estimated at more than USD 4 billion (24). Indirect costs such as reduced productivity, absenteeism, and long-term management of diabetes and cardiovascular disease further add to this economic burden. Emerging evidence also implicates environmental stressors, particularly endocrine-disrupting chemicals (EDCs) found in industrial pollutants, plastics, and pesticides, in exacerbating hormonal imbalances and ovarian dysfunction. These effects are especially pronounced in urban and industrialized settings (25). The use of varying diagnostic criteria, including those proposed by the Rotterdam consensus, the National Institutes of Health (NIH), and the Androgen Excess and PCOS Society (AES), further complicates efforts to standardize prevalence estimates across and within populations. This diagnostic variability hinders epidemiological surveillance and delays the formulation of targeted healthcare strategies. Addressing the multifaceted burden of PCOS necessitates a comprehensive and integrative approach. Standardized diagnostic protocols, enhanced public education, and equitable access to reproductive and metabolic healthcare, particularly in underserved rural regions, are critical priorities (18). Lifestyle interventions focusing on balanced nutrition, physical activity, and stress management remain cornerstone strategies in PCOS prevention and management (26). Integrating PCOS screening, education, and awareness campaigns into primary healthcare systems and school-based health programs could significantly improve early detection and long-term outcomes. Moreover, large-scale, population-based epidemiological studies are essential to refine global and regional prevalence estimates and to inform evidence-based policy development aimed at improving the health and quality of life of women with PCOS worldwide (17).

## 4 Pathobiology of PCOS

PCOS is a complex, multifactorial endocrine disorder in women, arising from the interplay of genetic susceptibility, environmental exposures, and modifiable lifestyle factors such as diet and physical activity. However, the precise etiology remains elusive; both hereditary influences and lifestyle-related contributors, including obesity and physical inactivity, are recognized as key determinants in the onset and progression of the syndrome (3). The pathophysiology of PCOS is principally marked by hormonal and metabolic dysregulation. Hyperandrogenism, a clinical hallmark feature of PCOS, is characterized by elevated circulating levels of androgens, which contribute to clinical manifestations such as hirsutism, acne, and androgenic alopecia (27), as given in **Table 1**. Another pivotal endocrine abnormality involves altered gonadotropin secretion, particularly an increased luteinizing hormone (LH) to follicle-stimulating hormone (FSH) ratio. This imbalance disrupts normal folliculogenesis, resulting in anovulation and menstrual irregularities. It also contributes to the formation of multiple small, fluid-filled follicles or “cysts” within the ovaries, leading to the characteristic polycystic ovarian morphology observed on ultrasonography. Insulin resistance is another critical component of PCOS pathogenesis (28). In affected individuals, peripheral tissues exhibit diminished sensitivity to insulin, prompting compensatory hyperinsulinemia. Elevated insulin levels exacerbate hyperandrogenism by stimulating ovarian theca cells to produce excess androgens, thereby intensifying endocrine dysfunction (29). Diagnostic criteria for PCOS—including those from the NIH (1990), Rotterdam consensus (2003), and the Androgen Excess and PCOS Society (2006)—differ in their requirements and thereby influence prevalence estimates. A detailed discussion of how these criteria affect global prevalence is provided in Section 3. Moreover, insulin resistance significantly elevates the risk of metabolic sequelae, including impaired glucose tolerance and type 2 diabetes mellitus. Obesity, particularly central or abdominal obesity, is frequently coexistent with PCOS and further amplifies insulin resistance and its associated reproductive and metabolic derangements. Clinically, PCOS presents with a broad spectrum of signs and symptoms, encompassing reproductive (e.g., anovulation, menstrual irregularity, infertility), dermatological (e.g., hirsutism, acne, alopecia), and metabolic (e.g., weight gain, dyslipidemia, insulin resistance) disturbances. Chronic health implications include an increased risk of type 2 diabetes, cardiovascular disease, and endometrial hyperplasia or cancer (30). In addition to hormonal and metabolic abnormalities, chronic low-grade inflammation and oxidative stress have been increasingly recognized as central to the pathophysiology of PCOS. Elevated levels of inflammatory markers, such as C-reactive protein (CRP), suggest a persistent pro-inflammatory state, which is thought to further impair insulin signaling and elevate cardiovascular risk (31). Oxidative stress is an imbalance between reactive oxygen species and antioxidant defenses, exacerbates ovarian dysfunction, and may contribute to systemic metabolic impairment, including effects on hepatic and adipose tissue function (32). The convergence of hormonal, metabolic, inflammatory, and oxidative mechanisms creates a self-reinforcing cycle that complicates disease progression and therapeutic management. Consequently, the effective management of PCOS necessitates a comprehensive and individualized approach. This typically involves lifestyle interventions such as dietary modification, physical activity, and weight management, pharmacologic therapies, and, where appropriate, complementary and non-pharmacologic treatments. Psychological support is also essential, given the emotional and mental health challenges frequently experienced by women with PCOS. Thus, due to its heterogeneous presentation and chronic course, PCOS often requires coordinated, multidisciplinary care tailored to the unique needs and goals of each patient (3).

**Table 1:** Common comorbidities associated with PCOS, its pathogenesis, and clinical aspects.

Clinical features	Comorbidities	Pathophysiology	Clinical impact
Metabolic syndrome	Insulin resistance	Impaired cellular response to insulin;	Increased androgen production Leading to compensatory hyperinsulinemia Risk for type 2 diabetes
	Pancreatic beta-cell dysfunction	Decreased insulin production	Hyperglycemia-induced various dysfunctions and organ failure
	Dyslipidemia	Abnormal lipid metabolism due to insulin resistance and androgen excess	Significant increase in various lipid markers: LDL, triglycerides; reduced HDL
	Obesity (increased BMI)	Associated with insulin resistance, dyslipidemia, and systemic inflammation abdominal obesity	Deteriorates hormonal imbalance, metabolic profile, and physical and mental well-being
	Non-Alcoholic Fatty Liver Disease (NAFLD)	Insulin resistance, dyslipidemia, and inflammation	Hepatic failure Increased risk of fibrosis Cirrhosis
	Others	Hypertension,	Increased risk for cardiovascular disease and mortality
Cardiovascular issues	Hypertension	Related to insulin resistance, inflammation, and endothelial dysfunction	Risk of CVDs and stroke
	Cardiovascular Disease (CVD)	Driven by chronic inflammation, oxidative stress, and dyslipidemia	Major cause of morbidity in PCOS
Reproductive problems	Infertility	Anovulation of irregular cycles from gonadotropin imbalance	Difficulty in conceiving without medical intervention
	Endometrial hyperplasia and cancer	Prolonged unopposed estrogen due to chronic anovulation	If untreated, there is an increased risk for cancer
Dermatological manifestations	Hirsutism	Hyperandrogenism- Excessive facial/body hair growth	Depression Low self-esteem
	Acne	Increased androgen levels	Persistent, often treatment-resistant acne
	Alopecia	Androgenic effects on hair follicles (scalp hair thinning)	Female-pattern hair loss
Psychological and mental insults	Depression & anxiety	Hormonal imbalance, body image issues, infertility-related stress	Decreased quality of life, increased mental health burden
	Body image disturbance & low Self-esteem	Hirsutism, obesity, and acne influence self-perception	Social withdrawal and reduced well-being
Inflammation	Chronic low-grade inflammation	Elevated CRP and pro-inflammatory cytokines	Insulin resistance and cardiovascular risk
Oxidative stress	Systemic oxidative damage leading to organ damage and organ failure	Imbalance between free radicals and antioxidants resulting in excess proxidants leading to damage of lipids, proteins, carbohydrates, and nucleic acid	Affects ovarian function, liver, and adipose tissue

The diagnosis of PCOS poses inherent challenges due to its diverse clinical manifestations and the overlap of its symptoms with other prevalent endocrine disorders. Over the past few decades, various diagnostic frameworks have been established, with the most widely adopted being the 1990 National Institutes of Health (NIH) criteria, the 2003 Rotterdam Consensus, and the 2006 Androgen Excess and PCOS Society (AES) criteria. The discrepancies among these guidelines have contributed to considerable variations in reported prevalence and clinical diagnoses across different populations and research studies. According to the NIH criteria, a PCOS diagnosis necessitates the presence of both chronic anovulation and clinical or biochemical evidence of hyperandrogenism, after excluding other underlying causes. The Rotterdam criteria, supported by the European Society for Human Reproduction and Embryology (ESHRE) and the American Society for Reproductive Medicine (ASRM), broadened the diagnostic scope by requiring any two of the following three features: oligo- or anovulation, clinical or biochemical signs of hyperandrogenism, and polycystic ovarian morphology observed via ultrasonography, again following the exclusion of alternative diagnoses. Conversely, the AES criteria prioritize hyperandrogenism as a fundamental diagnostic criterion, requiring either oligo-anovulation or polycystic ovarian morphology in addition. Scientifically, Polycystic ovarian morphology is typically characterized by the presence of 12 or more small follicles (2–9 mm in diameter) in each ovary or an increased ovarian volume exceeding 10 cm³. However, recent advancements in high-resolution ultrasound technology suggest refining these thresholds to 20 or more follicles per ovary (27). It’s crucial to recognize that ovarian morphology alone is not definitively indicative of PCOS and can be observed in up to one-third of healthy women without any clinical symptoms, particularly in adolescent and young adult populations.

Laboratory assessment in suspected PCOS involves measuring total and free testosterone, sex hormone-binding globulin (SHBG), dehydroepiandrosterone sulfate (DHEAS), and 17-hydroxyprogesterone to evaluate for hyperandrogenism and rule out late-onset congenital adrenal hyperplasia. Serum LH and FSH levels may reveal an elevated LH/FSH ratio, although this is not a mandatory diagnostic marker. Further evaluations, such as fasting glucose and insulin levels, lipid profile, and oral glucose tolerance testing, may be indicated, especially in individuals who are overweight or obese, to screen for associated metabolic abnormalities (3). Caution is warranted when diagnosing PCOS in adolescents, as physiological anovulation and acne are common during this developmental stage. Current recommendations for this age group emphasize the need for both persistent hyperandrogenism and menstrual irregularities extending beyond two years post-menarche for a presumptive diagnosis, with less emphasis placed on ultrasonography due to the frequent occurrence of multicystic ovaries in this population (28).

## 5 Integrative potential of natural bioactives in complementary therapeutics

Conventional medical treatments for PCOS primarily aim at symptom management rather than targeting the underlying pathophysiology of the disorder. In recent years, natural bioactives derived from plants, microbes, and animals have gained increasing attention as complementary and alternative therapeutic options due to their ability to influence hormonal, metabolic, and inflammatory pathways central to PCOS (33). Among these, polyphenols—particularly flavonoids—have emerged as promising therapeutic agents because of their antioxidant, anti-inflammatory, and hormone-regulating properties (34). These compounds contribute to ameliorating metabolic disturbances and chronic inflammation, thereby supporting hormonal equilibrium and addressing comorbid conditions such as type 2 diabetes mellitus, obesity, and cardiovascular disease (35). Plant-derived flavonoids are of special importance because they regulate hormonal activity, improve insulin sensitivity, and attenuate systemic inflammation, all of which are crucial in PCOS management (36). Several herbal sources rich in polyphenols and other natural bioactives have been investigated for their pharmacological effects in PCOS, as summarized in **Table 2**. Chamomile (*Chamomilla matricariae* L.), which contains apigenin, gallic acid, and tannins, has been reported to stimulate ovulation and alleviate oligomenorrhea and hirsutism while exerting anti-inflammatory and antioxidant effects (37, 38). Cinnamon (*Cinnamomum cassia* (L.) J. Presl), rich in terpenoids and glycosides, improves insulin sensitivity, regulates menstrual cycles, and modulates glucose metabolism (39, 40). Barberry (*Berberis aristata* Lindl.) provides berberine, which lowers leptin levels, improves insulin resistance, reduces oxidative stress, and supports lipid metabolism (41). Chaste tree (*Vitex agnus-castus* L.) contains agnuside and flavonoids that restore menstrual regularity and enhance fertility outcomes by modulating prolactin and estrogen balance (42, 43). Turmeric (*Curcuma longa* L.), which contains curcumin, luteolin, and apigenin, lowers androgen levels, increases estrogen, and provides strong antioxidant and anti-inflammatory effects (44, 45). Similarly, stinging nettle (*Urtica dioica* L.) supplies flavonoids, polyphenols, and sterols that reduce hirsutism, regulate inflammatory markers, and exert antioxidant activity (42). Other botanicals, such as spearmint (*Mentha* sp*icata* L.), fenugreek (*Trigonella foenum-graecum* L.), parsley (*Petroselinum crispum* (Mill.) Nym.), green tea (*Camellia sinensis* (L.) Kuntze), ginger (*Zingiber officinale* Roscoe), licorice (*Glycyrrhiza glabra* L.), black seed (*Nigella sativa* L.), pomegranate (*Punica granatum* L.), and raspberry (*Rubus idaeus* L.) have also been documented to exert diverse benefits including anti-androgenic effects, improved ovarian function, enhanced insulin sensitivity, reduced ovarian volume, lipid metabolism regulation, and restoration of endocrine balance (44, 46–49). Flavonoids, in particular, play a central role in PCOS by alleviating oxidative stress, which is a key contributor to ovarian dysfunction. Through neutralization of free radicals, they help preserve cellular integrity and ovarian function (50). Additionally, they regulate inflammatory signaling and lipid metabolism, thereby addressing insulin resistance and hyperlipidemia, both common metabolic disturbances in PCOS (51). Flavonoids also influence steroidogenesis, which is critical for maintaining androgen levels and supporting regular ovulation (52). Among these, apigenin and ellagic acid have attracted particular interest for their therapeutic relevance. Apigenin, found abundantly in chamomile, parsley, celery, and citrus fruits, possesses a unique chemical structure enabling strong antioxidant and anti-inflammatory actions. It has been reported to modulate ovarian function, reduce hyperandrogenism, and improve ovulatory outcomes (34, 38). Ellagic acid, naturally present in pomegranates, berries, and nuts, regulates lipid metabolism, reduces inflammation, lowers androgen levels, and enhances insulin sensitivity, while its potent antioxidant capacity counteracts oxidative stress and metabolic dysfunction (44, 53).

**Table 2:** Herbs/botanicals, natural bio-actives, and their pharmacodynamic action in PCOS management.

Herbs/ botanical	Major natural bioactive (s)	Pharmacodynamic action	References
Barberry (*Berberis aristata* Lindl.)	Berberine	✓ Lowers leptin and insulin resistance, ✓ Reduces oxidative stress, ✓ Supports lipid metabolism	(41)
Spearmint (*Mentha* sp*icata* L.)	Essential oils, flavonoids	✓ Anti-androgenic, ✓ Restores folliculogenesis	(44)
Chamomile (*Chamomilla matricariae* L.)	Apigenin, Gallic acid, Tannins	✓ Stimulates ovulation, ✓ Reduces oligomenorrhea and hirsutism, modulates oxidative stress, ✓ Anti-inflammatory	(37)
Chaste Tree (*Vitex agnus-castus* L.)	Agnuside, Flavonoids	✓ Restores menstrual regularity, ✓ Enhances fertility by modulating prolactin and estrogen balance	(42)
Ginger (*Zingiber officinale* Roscoe)	Gingerols, shogaols	✓ Anti-inflammatory, ✓ improves insulin resistance	(44)
Cinnamon (*Cinnamomum cassia* (L.) J. Presl)	Terpenoids, Glycosides	✓ Improves insulin sensitivity, ✓ Regulates menstrual cycles, ✓ Modulates glucose metabolism	(39)
Fenugreek (*Trigonella foenum-graecum* L.)	Saponins, alkaloids	✓ Improves menstrual cyclicity, ✓ Reduces ovarian volume	(44)
Parsley (*Petroselinum crispum* (Mill.) Nym.)	Apigenin	✓ Regulates ovarian function, ✓ Reduces androgen excess, ✓ Enhances insulin sensitivity, ✓ Antioxidant	(46)
Green tea (*Camellia sinensis* (L.) Kuntze)	Catechins	✓ Enhances insulin sensitivity, ✓ Reduces oxidative stress	(44)
Pomegranate (*Punica granatum* L.) Raspberry (*Rubus idaeus* L.)	Ellagic acid	✓ Regulates lipid metabolism, ✓ Lowers androgens, ✓ Reduces inflammation and oxidative stress	(47, 48)
Licorice (*Glycyrrhiza glabra* L.)	Glycyrrhizin, flavonoids	✓ Anti-androgenic, ✓ Modulates steroidogenesis	(44)
Stinging Nettle (*Urtica dioica* L.)	Flavonoids, Polyphenols, Sterols	✓ Anti-inflammatory, ✓ Antioxidant, ✓ Reduces hirsutism, ✓ Modulates inflammatory markers	(49)
Black seed (*Nigella sativa* L.)	Thymoquinone	✓ Restores ovarian structure, ✓ Improves endocrine balance	(44)
Turmeric (*Curcuma longa* L.)	Curcumin, Luteolin, Apigenin	✓ Lowers androgen levels, ✓ Increases estrogen, ✓ Balances hormones, ✓ Anti-inflammatory ✓ Antioxidant	(45)

Taken together, these findings highlight that natural bioactives, particularly flavonoids such as apigenin and ellagic acid, provide multifaceted benefits in PCOS by targeting hormonal imbalance, metabolic dysfunction, and chronic inflammation. Their integrative use alongside conventional therapies represents a promising approach for more holistic and personalized management of PCOS (35).

## 6 Therapeutic potential and mechanistic insights

Apigenin and ellagic acid are naturally occurring polyphenols with significant therapeutic potential in polycystic ovary syndrome (PCOS) (15, 54). Both act through multi-targeted mechanisms—anti-inflammatory, antioxidant, anti-androgenic, and insulin-sensitizing effects—thereby addressing the endocrine, metabolic, and inflammatory disturbances characteristic of the disorder (55). Apigenin improves ovarian steroidogenesis and insulin sensitivity via PI3K/Akt signaling (56), while ellagic acid reduces oxidative stress and inflammation through ROS scavenging, NF-κB/TNF-α suppression, and 5α-reductase inhibition, in parallel regulating glucose- and lipid-metabolizing enzymes to restore metabolic balance (57, 58). Beyond these established actions, both compounds target emerging PCOS-related pathways. They alleviate mitochondrial dysfunction (apigenin via PGC-1α/Nrf2-mediated biogenesis; ellagic acid through stabilization of mitochondrial membrane potential and ATP enhancement), inhibit NLRP3 inflammasome activation, and restore gut microbial diversity, including butyrate-producing taxa, thereby reducing endotoxin-driven inflammation and improving the gut–ovary axis (59). While usually studied independently, apigenin and ellagic acid exhibit complementary and potentially synergistic effects by enhancing antioxidant defenses, improving insulin sensitivity, regulating ovarian function, and suppressing hyperandrogenism. Collectively, these actions underscore their promise as natural adjunctive agents in PCOS management, supporting future clinical trials of combinatorial and personalized interventions (see **Table 3**).

**Table 3:** Emerging molecular pathways in PCOS and modulatory effects of apigenin and ellagic acid.

Pathway/mechanism	Role in PCOS pathology	Effect of apigenin	Effect of ellagic acid	References
NLRP3 inflammasome	Promotes ovarian inflammation, IL-1β secretion, insulin resistance	Inhibits NLRP3 activation via NF-κB suppression; reduces IL-1β release	Suppresses ROS-mediated NLRP3 activation; reduces caspase-1 activity	(14, 65)
Gut–ovary axis	Dysbiosis increases endotoxemia, inflammation, metabolic derangements	Restores microbial diversity; enhances SCFAs; lowers endotoxin leakage	Promotes growth of beneficial microbes (e.g., Bifidobacterium, Akkermansia)	(66, 67)
Mitochondrial dysfunction	Impairs oocyte quality, increases ROS, disrupts energy metabolism	Activates PGC-1α and Nrf2 pathways; improves mitochondrial biogenesis and function	Stabilizes mitochondrial membrane potential; reduces ROS; enhances ATP production	(68, 69)
Oxidative stress–inflammation loop	Drives systemic inflammation, worsens insulin resistance and androgen excess	Strong antioxidant activity via Nrf2 activation; scavenges free radicals	Potent ROS scavenger; upregulates antioxidant enzymes (SOD, catalase, GPx)	(12, 70)

## 7 Comparative effectiveness of apigenin and ellagic acid vs. standard drugs

### 7.1 Apigenin vs. standard drugs

Natural flavonoids like apigenin have shown anti-inflammatory, hormone-modulating, and antioxidant benefits that are pertinent to PCOS (60). Common PCOS medications, such as metformin and clomiphene citrate, mainly target ovulation induction and insulin resistance, respectively (61). Apigenin may improve the hormonal balance in PCOS by dramatically lowering estrogen and testosterone levels while raising progesterone and FSH levels, according to research conducted in animal models (55). Standard hormonal therapies, such as oral contraceptives, manage hormone levels, although they may have distinct processes and adverse effect profiles (62). Similar to metformin, apigenin has demonstrated potential in enhancing antioxidant status and lipid profiles in PCOS rat models. Metformin is a well-known medication for treating insulin resistance and related metabolic issues in PCOS (60, 63). Apigenin has been shown to increase superoxide dismutase activity and overall antioxidant capacity while decreasing pro-inflammatory cytokines (TNF-α, IL-6) and total oxidative state. PCOS is frequently associated with oxidative stress and persistent low-grade inflammation, both of which may be treated by conventional medications, but sometimes in different ways (55, 64). Apigenin may have fewer negative effects than synthetic medications due to its natural composition. However, this must be extensively studied in clinical studies.

### 7.2 Ellagic acid vs. standard drugs

A polyphenolic substance found in large quantities in pomegranates, berries, and nuts, ellagic acid has significant anti-inflammatory and antioxidant properties, which makes it a promising therapy adjunct for PCOS. There are similarities and differences when compared to common medications like letrozole, clomiphene citrate, and metformin. Similar to metformin, ellagic acid has a positive modulation of lipid profiles, lowers fasting blood glucose, and dramatically increases insulin sensitivity. However, ellagic acid also has direct anti-androgenic effects by restricting 5α-reductase, decreasing circulating testosterone levels. This trait is not shared by metformin, which primarily targets insulin resistance. In contrast to ovulation-inducing drugs like letrozole and clomiphene, ellagic acid restores follicular dynamics via reducing oxidative stress and restoring normal ovarian morphology, rather than directly altering estrogen receptors. This mechanism suggests a possible function for ellagic acid as an adjuvant to conventional ovulation medications, as it enhances rather than replicates the action of selective estrogen receptor modulators. Evidence from clinical trials supports these conclusions more thoroughly. In a randomized, double-blind, placebo-controlled study, women with PCOS were given 200 mg of ellagic acid daily for eight weeks (14; n = 60). Improvements in metabolic and hormonal parameters were statistically significant when compared to a placebo. In particular, ellagic acid decreased serum total testosterone (−0.6 ± 0.2 ng/mL; p < 0.05), fasting insulin (−3.5 ± 1.1 μIU/mL; p < 0.05), fasting blood sugar (−11.2 ± 3.6 mg/dL; p < 0.01), and Homeostatic Model Assessment of Insulin Resistance (HOMA-IR index) (−1.3 ± 0.4; p < 0.01). While HDL showed an apparent but nonsignificant rise, lipid markers such as total cholesterol (−15.4 ± 4.2 mg/dL; p < 0.05) and LDL cholesterol (−12.7 ± 3.9 mg/dL; p < 0.05) also improved dramatically. The levels of pro-inflammatory cytokines, such as TNF-α and IL-1β, were decreased (p < 0.01). According to these findings, ellagic acid has distinct anti-inflammatory and androgen-lowering qualities in addition to similarities with metformin’s metabolic advantages, offering a wider range of therapeutic options for the treatment of PCOS (65–70).

Standard pharmacological treatments such as oral contraceptive pills (OCPs), metformin, clomiphene citrate, and letrozole remain first-line options for PCOS (71); however, these primarily target symptomatic relief rather than addressing the underlying pathophysiological mechanisms, and their long-term use may be associated with adverse effects. In contrast, apigenin and ellagic acid demonstrate multi-targeted actions by simultaneously modulating insulin resistance, hyperandrogenism, oxidative stress, inflammation, and broader metabolic dysfunctions. Unlike OCPs or selective estrogen receptor modulators, which focus mainly on reproductive hormone regulation, these natural bioactives also exert antioxidant, mitochondrial-protective, and gut–ovary axis–modulating effects, thereby offering wider systemic benefits. Although their clinical evidence base is less extensive than that of standard drugs, their favorable safety profiles, broad mechanistic actions, and potential for synergistic use with conventional therapies highlight their promise as integrative or adjunctive agents in PCOS management (72). A comparative summary (**Table 4**) underscores the distinctions between apigenin, ellagic acid, and standard therapies in terms of molecular targets, therapeutic effects, evidence base, and side-effect profiles. Notably, while ellagic acid is less extensively studied than apigenin, emerging data suggest it may be particularly effective in reducing inflammation and hyperandrogenism while achieving comparable metabolic regulation to standard medications. Nonetheless, conclusive comparative effectiveness, especially against ovulation-inducing drugs and metformin, requires rigorously designed head-to-head clinical trials.

**Table 4:** Comparative evaluation of apigenin and ellagic acid versus standard therapies in PCOS management.

Agent/class	Primary targets & mechanisms	Key benefits in PCOS	Limitations/ risks	Evidence base	Clinical trial status
Oral Contraceptives (OCPs)	Suppress LH → ↓androgen production; regulate cycles	Reduces hirsutism, acne, and restores menstrual regularity	No effect on insulin resistance; ↑ risk of thromboembolism, metabolic side effects	Extensive RCTs; guideline-recommended	Extensive RCTs; robust long-term human data; guideline-recommended first-line therapy
Metformin (Biguanide)	AMPK activation; ↓hepatic gluconeogenesis; ↑glucose uptake	Improves insulin sensitivity, menstrual cyclicity, and modest androgen reduction	GI intolerance, B12 deficiency, and rare lactic acidosis	Strong RCT support; standard for metabolic PCOS	Robust RCTs (multiple large-scale human trials)
Clomiphene Citrate (SERM)	ER antagonism → ↑GnRH → ↑FSH/LH	Induces ovulation, improves fertility	Resistance in some patients; multiple pregnancy risk	Established fertility therapy	Multiple RCTs, guideline-recommended
Letrozole (Aromatase Inhibitor)	Blocks aromatase → ↓estrogen → ↑FSH	More effective than clomiphene for ovulation and live births	Arthralgia, fatigue; teratogenic risk	Multiple RCTs; first-line ovulation induction	Robust RCTs
Spironolactone/Anti-androgens	Blocks androgen receptors	Reduces hirsutism, acne	Teratogenic; menstrual irregularities; long-term safety concerns	Clinical use common; evidence moderate	Limited RCTs, mostly observational
Apigenin (Flavone)	PI3K/Akt↑, AMPK↑, NF-κB↓, CYP17A1↓, NLRP3↓	Improves insulin sensitivity; ↓inflammation and oxidative stress; modulates steroidogenesis; supports folliculogenesis	Limited clinical data; poor bioavailability	Robust preclinical data; few small trials	Early-stage trials; limited human data
Ellagic Acid (Polyphenol)	NF-κB↓, AMPK↑, 5α-reductase↓, Nrf2↑, mitochondrial protection	↓testosterone, improves lipid/glucose profile; ↓inflammation; restores ovarian morphology; microbiome modulation	Limited human studies; gut microbiome-dependent metabolism	Preclinical + small RCTs show metabolic & hormonal benefits	Small RCTs

Beyond clinical outcomes, cost is an important factor in PCOS management. Conventional therapies such as metformin and letrozole are inexpensive generic drugs but require long-term use and monitoring for side effects, which adds to healthcare costs. In contrast, natural bioactives like apigenin and ellagic acid are derived from dietary sources (parsley, chamomile, pomegranates, berries), making them relatively accessible and affordable as nutraceuticals. Although standardized formulations may increase costs initially, their favorable safety profiles and potential to reduce polypharmacy could enhance long-term cost-effectiveness in integrative approaches. However, formal pharmacoeconomic analyses comparing these agents to standard drugs are currently lacking and should be prioritized in future research.

## 8 Molecular targets and signaling pathways

While Section 6 outlined the broad systemic mechanisms, this section focuses on the specific molecular targets regulated by apigenin and ellagic acid. Key intracellular cascades implicated in PCOS pathogenesis include PI3K/Akt, NF-κB, AMPK, CYP17A1, and Nrf2. By modulating these pathways, apigenin and ellagic acid address core features of the disorder such as insulin resistance, hyperandrogenism, oxidative stress, and chronic inflammation. Dysregulation of PI3K/Akt signaling underlies insulin resistance in PCOS. In preclinical models, apigenin restores insulin sensitivity and ovarian function by inhibiting PI3K/Akt and downstream mTOR activation, thereby reducing excessive follicular growth and anovulation (73). Chronic inflammation in PCOS is driven by NF-κB–mediated transcription of pro-inflammatory cytokines such as TNF-α and IL-6; both compounds suppress NF-κB activation, lowering systemic and ovarian inflammation (65). Ellagic acid further improves metabolic homeostasis by stimulating AMPK, which enhances glucose uptake, lipid oxidation, and energy balance (73). Apigenin also inhibits CYP17A1, a key enzyme in androgen biosynthesis, thereby attenuating hyperandrogenism and lowering circulating testosterone levels (74). Through coordinated modulation of these interlinked pathways, apigenin and ellagic acid exert multi-targeted therapeutic effects, reinforcing their potential as natural agents for managing both the endocrine and metabolic dimensions of PCOS.

## 9 Toxicological and safety data

Experimental research indicates that apigenin is neither mutagenic nor genotoxic and is widely regarded as safe, given its broad occurrence in dietary sources such as parsley, chamomile, and celery (75). Similarly, ellagic acid, found in pomegranates, berries, and nuts, is considered safe at dietary levels. Most available safety data, however, are derived from animal studies. High doses of ellagic acid (≥200 mg/kg) have been associated with renal and cardiac toxicity in rats, while reported LD50 values vary across models (76). Apigenin also demonstrates a favorable safety profile in rodents, with several studies reporting no observed adverse effects at nutritionally relevant doses (77). Importantly, NOAEL values for apigenin and ellagic acid have not yet been established in human clinical studies. Current human trials have primarily evaluated short-term supplementation and have not systematically assessed dose–toxicity relationships. This gap highlights the need for carefully designed safety studies in humans before therapeutic dosing ranges can be standardized for PCOS management. Another practical consideration is the potential for herb–drug interactions. Both apigenin and ellagic acid undergo extensive metabolism via cytochrome P450 (CYP450) enzymes and phase II conjugation pathways. Apigenin, for example, has been reported to inhibit CYP3A4 and CYP2C9, raising the possibility of altered pharmacokinetics when co-administered with oral contraceptives, statins, or antidiabetic drugs (78). Ellagic acid is subject to gut microbiota–mediated metabolism to urolithins, which may interact with drugs affecting intestinal absorption or hepatic clearance. While clinically significant interactions have not been reported in PCOS trials, caution is warranted in integrative practice, and future studies should specifically evaluate safety in the context of polypharmacy.

## 10 Microbiome and gut-ovary axis

The pathophysiology of PCOS is now understood to be significantly influenced by the complex interaction between the gut microbiota and the ovaries, known as the gut-ovary axis (66). Women with PCOS have often been shown to have dysbiosis, which is defined as an imbalance in the gut microbiota’s composition and activity (79). Altered gut microbiota may increase intestinal permeability, allowing metabolites like LPS into circulation, triggering chronic inflammation and insulin resistance—key features of PCOS (80). For example, in animal models, ellagic acid, a polyphenol included in fruits and nuts, has been shown to decrease the prevalence of pro-inflammatory bacteria while increasing the development of beneficial bacteria such as *Bifidobacterium* species and *Akkermansia muciniphila* (67). Likewise, it has been demonstrated that apigenin, a flavonoid found in many plants, has a beneficial effect on the variety and composition of gut microbes, which may lessen metabolic abnormalities linked to dysbiosis (81). More study is needed to completely understand the particular processes by which ellagic acid and apigenin modify the gut microbiota and how these changes translate into enhancements in ovarian function and metabolic health in women with PCOS.

## 11 Discussion on nano delivery or formulation strategies

The therapeutic use of apigenin and ellagic acid is limited by poor oral bioavailability due to low solubility, rapid metabolism, and restricted permeability (82). Nanotechnology-based carriers such as nanoparticles, liposomes, and phytosomes have been developed to address these barriers by improving solubility, stability, and targeted delivery (83–87). Preclinical studies on related polyphenols demonstrate their potential: quercetin nanoparticles showed enhanced antioxidant activity (88), liposomal silymarin improved hepatoprotective efficacy, and phytosomal curcumin exhibited greater absorption (89). For apigenin, nanoformulations such as solid lipid and polymeric nanoparticles have enhanced stability, uptake, and antioxidant effects in metabolic models (88), while ellagic acid phytosome and nano-liposome formulations improved systemic exposure and insulin sensitivity in metabolic dysfunction models (90, 91). However, PCOS-specific evidence remains scarce, and translation to clinical application is hindered by high production costs, formulation variability, and the need for rigorous toxicological evaluation. Even so, these delivery platforms offer a promising strategy to enhance the therapeutic efficacy of apigenin and ellagic acid in PCOS and related comorbidities. Future research should prioritize PCOS-specific nanoformulation trials and clinical translation to fully realize the therapeutic potential of these compounds. To summarize these considerations, **Table 5** outlines the main delivery systems investigated for apigenin and ellagic acid, highlighting their potential benefits and current research status.

**Table 5:** Nano-delivery approaches for apigenin and ellagic acid.

Delivery system	Compound	Reported advantages	Research status (PCOS-specific vs. general)	Challenges	References
Phytosomes	Ellagic acid	↑ Solubility, ↑ oral absorption, improved bioavailability	Tested in metabolic dysfunction models; not yet in PCOS	Cost of production, variability in formulations	(84, 90)
Liposomes	Ellagic acid, Apigenin	Biocompatible, protect from degradation, enable targeted delivery	Animal studies in oxidative stress/metabolic syndrome; no PCOS-specific data	Stability issues, regulatory hurdles	(85, 89)
Solid lipid nanoparticles (SLN)	Apigenin	Enhanced antioxidant activity, improved stability and uptake	Demonstrated in metabolic models; not yet tested in PCOS	Scalability, higher production costs	(68, 83)
Polymeric nanoparticles	Apigenin	Controlled release, improved cellular uptake	*In vitro* and *in vivo* (non-PCOS) studies; translation to PCOS awaited	Regulatory approval, long-term safety data lacking	(81, 91)
Nanocrystals/co-crystals	Apigenin & Ellagic acid	↑ Dissolution rate, improved pharmacokinetics	Early-stage studies; no PCOS trials yet	Limited human data, scalability concerns	(76, 88)

## 12 Preclinical and clinical studies

**Figure 1:**
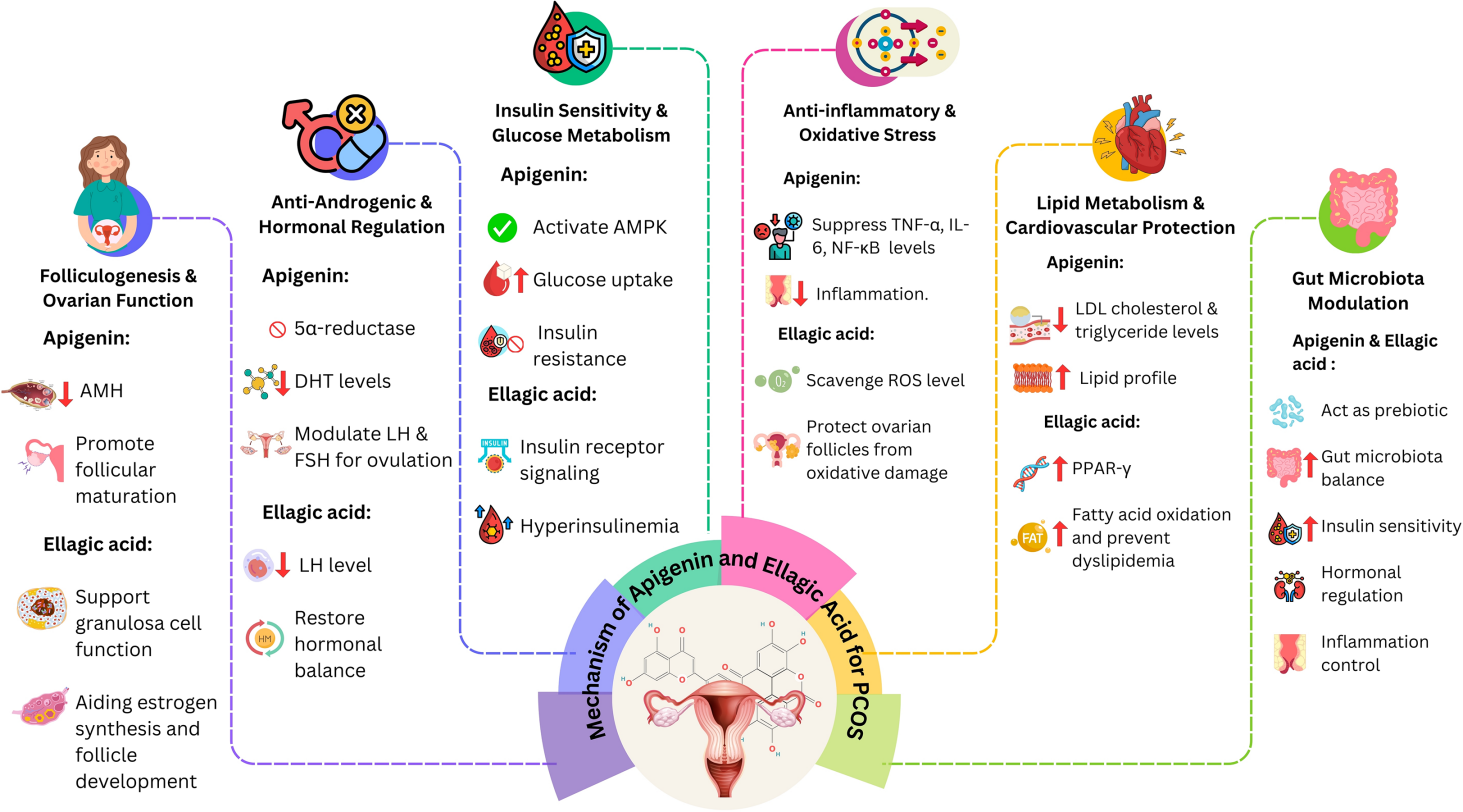
Mechanistic insights of apigenin and ellagic acid for PCOS management.

Numerous 0preclinical experimental (animal) and clinical (human) studies have investigated the pharmacological and toxicological effects of natural bioactives in the management of PCOS. Berk et al. (92) reported that apigenin exerts protective effects against PCOS in female Wistar albino rats by reducing oxidative stress, body weight, and the levels of progesterone, FSH, LH, as well as the LH/FSH ratio. Additionally, apigenin suppresses inflammatory mediators, including IL-1β, IL-13, and IL-18. Similarly, Darabi et al. demonstrated that apigenin significantly decreased levels of androgenic hormones (including estrogen, testosterone, LH, and the LH/FSH ratio) and inflammatory cytokines (TNF-α and IL-6) in a PCOS-induced Wistar rat model (55). This was accompanied by an increase in total antioxidant levels and superoxide dismutase (SOD) activity, leading to a reduction in ovarian cysts and theca layer thickness, while improving the corpora lutea and granulosa layer thickness. These effects were associated with the downregulation of NF-κB transcriptional activity. Peng et al. further supported these findings in a study using Sprague Dawley rats, where apigenin treatment improved lipid profiles, enhanced antioxidant levels, and elevated estradiol concentrations, while suppressing TNF-α, IL-6, ovarian cyst diameter, and the thickness of granulosa and theca layers. (60) In addition to flavonoids like apigenin, plant-based therapies have also shown promise. *Tetracera potatoria*, a plant known for its antioxidant, anti-inflammatory, and hormone-balancing properties, demonstrated beneficial effects against PCOS in female Wistar rats (93). Galati et al. conducted a study on Sprague Dawley rats using a PCOS model and observed that treatment led to reduced body weight, improved lipid profiles, decreased cyst diameter, and restoration of gonadotropin hormones such as estradiol and testosterone (94). The treatment also supported follicular health by suppressing TNF-α and IL-6 levels. Another promising natural bioactive is ellagic acid (EA), which possesses both antioxidant and anti-inflammatory properties. In a PCOS model induced by estradiol valerate in mice, EA reversed elevated LH levels, normalized miRNA-21 expression, and restored the number of primordial and Graafian follicles (95, 96). Moreover, EA treatment improved ovarian morphology, reduced theca layer thickness, enhanced the oocyte layer, and improved the quality of antral and preovulatory follicles (**Figure 1**). Additionally, a placebo-controlled, randomized, double-blind clinical trial was conducted to assess the therapeutic efficacy of EA in patients with PCOS (14). The study involved 60 participants, who were randomly assigned to receive 200 mg/day of EA for 8 weeks. Throughout the trial, daily blood samples were collected at night, and 95% patient compliance was reported. After the study, no significant change in body weight was observed. However, patients who received EA demonstrated notable reductions in fasting blood sugar (FBS), insulin levels, lipid profile markers, including total cholesterol (TC), triglycerides (TG), and low-density lipoprotein (LDL), as well as inflammatory mediators, specifically TNF-α and IL-1β, and insulin resistance indices. Moreover, EA supplementation resulted in a significant increase in the persistence of beneficial gut bacteria and a reduction in oxidative stress. Hormonally, EA-treated patients showed decreased levels of total testosterone, prolactin (PRL), and anti-Müllerian hormone (AMH), while levels of FSH and LH remained unchanged compared to baseline values. In another clinical trial, EA supplementation was examined in 12 female patients with metabolic syndrome led to a significant reduction in waist circumference (from 102.2 ± 4.2 cm to 99.5 ± 3.2 cm, *p* < 0.05). Other improvements, such as lower blood pressure, triglycerides, fasting glucose, insulin, and enhanced insulin sensitivity, were observed in the overall cohort (97). These findings underscore the potential role of ellagic acid, along with apigenin, as effective complementary agents in the management of PCOS. Further preclinical and clinical studies are warranted to elucidate their mechanisms of action and confirm their therapeutic value in human populations affected by PCOS.

## 13 Pharmacokinetics, bioavailability, and endocrine modulation

EA, a natural bioactive present in ellagitannin-rich foods (98), undergoes digestion beginning in the stomach and absorption in the jejunum and ileum (99). Its metabolism is largely gut microbiota–dependent, producing bioactive urolithins such as urolithin A, dihydroxy urolithin A, and urolithin B (90, 100). These metabolites undergo hepatic phase I (hydroxylation) and phase II transformations (methylation, sulfonation, glucuronidation), enhancing solubility and promoting urinary excretion (101, 102). EA and its derivatives distribute across muscle, adipose tissue, heart, and lungs (103), where they persist for 24–48 hours due to enterohepatic recycling (104, 105). While apigenin, a flavone abundant in parsley, chamomile, and celery, also demonstrates low oral bioavailability, attributed to poor solubility and extensive first-pass metabolism (78). It is absorbed along the gastrointestinal tract via active transport in the duodenum/jejunum and passive diffusion in the ileum/colon (106), undergoing hydrolysis and glucuronidation (91, 107, 108). Once in circulation, apigenin binds serum transferrin and distributes to the liver, kidney, and intestines (109). It undergoes phase I metabolism via CYP450 and FMO enzymes, and phase II via sulfonation and glucuronidation, producing β-monoglucuronides (94, 110, 111). Ultimately, it is excreted through urine, with residual elimination via feces, partly influenced by gut microbiota (112).

Both apigenin and EA face the challenge of poor oral bioavailability, but advanced formulation strategies are showing promise. Nanoparticles, solid lipid nanoparticles, nanocrystals, liposomes, and phytosomes improve stability, solubility, and targeted delivery (76, 84, 85, 88). Innovative approaches such as prodrug design and co-crystal technology further enhance pharmacokinetics (83). Moreover, modulation of gut microbiota, which plays a key role in EA metabolism to urolithins, represents an emerging strategy to boost efficacy (67, 90, 100). Beyond pharmacokinetics, both compounds exhibit endocrine modulation relevant to PCOS. Apigenin restores LH/FSH balance, inhibits CYP17A1 to reduce androgen excess, and enhances progesterone and FSH levels, thereby supporting folliculogenesis and ovulation (55, 68, 113). Ellagic acid lowers testosterone and dihydrotestosterone (DHT) via 5α-reductase inhibition and antioxidant activity, while indirectly modulating estrogen–progesterone feedback loops (14, 114). Collectively, these mechanisms normalize gonadotropin signaling, alleviate hyperandrogenism, and restore menstrual cyclicity. Unlike OCPs, which suppress the hypothalamic–pituitary–ovarian (HPO) axis pharmacologically, apigenin and ellagic acid modulate endocrine function physiologically, suggesting potential for safer long-term reproductive outcomes (62). Taken together, advances in formulation strategies and a deeper understanding of endocrine modulation highlight the potential of apigenin and ellagic acid as clinically viable therapeutics. Future research should integrate nanodelivery systems, prodrugs, and microbiota-targeted approaches with rigorous endocrine profiling in clinical trials to optimize their role in PCOS management (82, 86).

## 14 Constraints of existing evidence

Despite promising preclinical and early clinical findings, several limitations constrain the translation of apigenin and ellagic acid into mainstream PCOS therapy. A major hurdle lies in regulatory classification. In the United States, the FDA regulates polyphenols marketed as supplements under the Dietary Supplement Health and Education Act (DSHEA, 1994), which restricts claims to general “structure–function” benefits (e.g., “supports metabolic health”). Any disease-specific indication, such as PCOS, would require progression through the Investigational New Drug (IND) and New Drug Application (NDA) pathways, with robust randomized controlled trials. In Europe, the European Food Safety Authority (EFSA) requires formal evaluation and approval of health claims under Regulation (EC) No 1924/2006, while nanoformulations or novel delivery systems often fall under the Novel Food Regulation (EU 2015/2283), requiring comprehensive safety and toxicological data. These frameworks mean that standardized nanoformulations of apigenin or ellagic acid would need substantially more data than conventional plant extracts to meet regulatory approval. Another key limitation is bioavailability. Both compounds undergo rapid metabolism and poor absorption, limiting systemic exposure. While nano-delivery systems (e.g., phytosomes, liposomes, solid lipid nanoparticles) have shown improved pharmacokinetics in metabolic and oxidative stress models, no PCOS-specific nanoformulation trials have yet been reported. From a practical perspective, widespread clinical use depends on the balance between benefit and cost. Nanoformulations are considerably more expensive to manufacture and scale compared to conventional extracts, with added challenges of stability and regulatory toxicology (115). Nevertheless, if such formulations can demonstrably enhance exposure two- to three-fold, reduce dosing frequency, or minimize adverse effects relative to standard therapy, they may ultimately prove cost-effective by lowering reliance on multiple drugs and reducing downstream healthcare costs. Rigorous pharmacoeconomic studies will therefore be essential to establish their real-world feasibility.

## 15 Research gaps and roadmap for clinical translation

Although apigenin and ellagic acid demonstrate strong promise in preclinical and limited clinical settings, critical research gaps remain before they can be integrated into evidence-based management of PCOS. Future trials should incorporate validated metabolic and endocrine biomarkers to better define treatment responses. Key candidates include HOMA-IR to assess insulin resistance, serum testosterone and sex hormone–binding globulin (SHBG) for androgen excess, anti-Müllerian hormone (AMH) to reflect ovarian reserve and follicular activity, and inflammatory cytokines such as TNF-α and IL-6. Integrating these biomarkers with traditional clinical measures such as menstrual cyclicity and ovulation rates would provide deeper mechanistic insights and allow phenotype-specific efficacy assessments. Another important research direction is the evaluation of synergistic or add-on therapeutic strategies. Since apigenin and ellagic acid modulate complementary pathways including AMPK activation, NF-κB inhibition, and CYP17A1 or 5α-reductase suppression, their combined use may provide broader benefits than either compound alone. Moreover, combining these bioactives with standard therapies such as metformin or letrozole has the potential to enhance outcomes while reducing required drug doses, thereby minimizing adverse effects. Beyond biochemical and mechanistic endpoints, future clinical trials should align more closely with patient priorities by incorporating quality-of-life measures, psychological well-being assessments, and fertility-related outcomes such as ovulation, conception, and live birth rates. These endpoints capture the multidimensional burden of PCOS and ensure that therapeutic benefits are meaningful in real-world contexts. Collectively, the roadmap toward clinical translation should include systematic human safety and pharmacokinetic studies, biomarker-driven and phenotype-stratified randomized controlled trials, rigorous exploration of combination regimens, and incorporation of patient-centered outcomes. Such an approach would not only strengthen the clinical evidence base but also facilitate regulatory acceptance and support cost-effective integration of apigenin and ellagic acid into PCOS management.

## 16 Conclusions

Apigenin and ellagic acid represent promising adjuncts for the integrative management of PCOS, with evidence suggesting benefits across metabolic, endocrine, and inflammatory pathways. Moving forward, emphasis should shift from reiterating preclinical challenges toward designing rigorous, biomarker-driven randomized controlled trials that can define their true clinical value. Such trials should consider phenotype-stratified populations—for example, insulin-resistant versus lean hyperandrogenic subgroups—to determine whether responses differ across the heterogeneous PCOS spectrum. Combination strategies also warrant exploration, including co-administration of apigenin and ellagic acid or their use alongside standard therapies such as metformin or letrozole to enhance efficacy and reduce drug-related side effects. Importantly, future research should adopt a personalized medicine framework, integrating molecular biomarkers, patient-centered outcomes, and cost-effectiveness analyses. This precision approach would not only advance the clinical translation of these bioactives but also ensure that therapies are tailored to the diverse presentations and priorities of women living with PCOS.
